# Glycogen storage disease type Ia misdiagnosed as multiple acyl-coenzyme A dehydrogenase deficiency by mass spectrometry

**DOI:** 10.3389/fped.2022.999596

**Published:** 2022-11-14

**Authors:** Juan Du, Li-Min Dou, Yong-Hong Jin, Qing-Fen Wen, Ya-Fen Lin, Jian-She Wang

**Affiliations:** ^1^ Department of Pediatrics, Jinshan Hospital of Fudan University, Shanghai, China; ^2^ The Center for Pediatric Liver Diseases, Children’s Hospital of Fudan University, Shanghai, China

**Keywords:** glycogen storage disease type Ia, glucose-6-Phosphatase, multiple acyl- coenzyme a dehydrogenase deficiency, mass spectrometry, gene variant

## Abstract

**Objective:**

To report a case of glycogen storage disease (GSD) type Ia misdiagnosed as multiple acyl-coenzyme a dehydrogenase deficiency (MADD) by mass spectrometry.

**Methods:**

A 7 months old boy was admitted to our hospital for elevated transaminase levels lasting more than 1 month. His blood biochemistry showed hypoglycemia, metabolic acidosis, hyperlipidemia, elevated lactate and uric acid, elevated alanine amino transferase (ALT), aspartate amino transaminase (AST) and gamma-glutamyl transferase (GGT). Mass spectrometry analysis of blood and urine showed elevated blood acylcarnitines and dicarboxylic aciduria, indicating multiple acyl-coenzyme A dehydrogenase deficiency. Sanger sequencing of all exons of glucose-6-phosphatase (G6Pase) and electronic transfer flavoprotein dehydrogenase (ETFDH) was performed for the patient and his parents.

**Results:**

Coding and flanking sequences of the G6Pase gene detected two heterozygous single base substitutions in the boy. One variant was in exon 1 (c.209G > A), Which was also detected in the father. Another was in exon 5 (c.648G > T), which was detected in the mother. Coding and flanking sequences of the ETFDH gene revealed no pathogenic/likely pathogenic variants in the boy.

**Conclusion:**

GSD Ia can manifest elevated blood acyl carnitines and dicarboxylic aciduria which were the typical clinical manifestations of MADD. So the patient with clinical manifestations similar to MADD is in need of differential diagnosis for GSD Ia. Genetic testing is helpful to confirming the diagnosis of inherited metabolic diseases.

## Introduction

Glycogen storage disease (GSD) type Ia (OMIM#232200) is a glycogen metabolism disorder due to a gene defect in glucose-6-phosphatase (G6Pase). This disease is autosomal recessive with typical clinical features of hypoglycemia, an enlarged liver, growth retardation and a tendency to bleed, accompanied by hyperlipidemia, hyperuricemia, and hyperlactatemia. Long-term complications of this disease can include gout, hepatocellular adenoma, platelet dysfunction, renal insufficiency, osteoporosis and others (1). Multiple acyl-coenzyme A dehydrogenase deficiency (MADD) (OMIM#231680) is a fatty acid oxidation metabolic disorder caused by a gene defect in mitochondrial electron transport flavoprotein (ETF) or electronic transfer flavoprotein-ubiquinone oxidoreductase [ETF-QO, also known as electronic transfer flavoprotein dehydrogenase (ETFDH)]. MADD is an autosomal recessive disease with multiple clinical features, predominantly intermittent myasthenia; however, other symptoms such as drowsiness, vomiting, hypoglycemia, metabolic acidosis, and enlarged liver during acute attacks may also be present. GSD Ia shares similar clinical manifestations with MADD, such as hypoglycemia, enlarged liver, elevated liver transaminase, and metabolic acidosis. GSD Ia is rarely reported with abnormal carnitine and/or abnormal urine organic acid levels. A child patient was admitted to the pediatric department of Jinshan Hospital Fudan University in July 2012 with suspicious MADD as diagnosed by mass spectrometry and was eventually diagnosed with GSD Ia by genetic studies. The case is reported below.

## Materials and methods

The boy (7 months old) was admitted to our hospital for elevated transaminase levels lasting more than 1 month. One month previously, he was admitted to a local hospital due to bronchopneumonia. During the stay, elevated transaminases were occasionally revealed by laboratory tests. He was referred to us for persistently elevated transaminases during multiple follow-ups and the reappearance of cough and fever two days previously. The child was G1P1 and was born at full-term with a birth weight of 2,290 g. He was once hospitalized for “neonatal hypoglycemia and hyperbilirubinemia” in another hospital with detailed information unavailable. The patient received mixed feeding after his discharge. He drank milk more frequently and appeared easy hungry than the common infant. The child was given solid food supplementation from 4 months and rice supplementation from 7 months, which he liked. The child could raise his head in the third month of age but still could not climb or sit alone at presentation. Both parents of the child were healthy and were in a non-consanguineous marriage with no history of genetic disease.

Physical examination at admission showed a body temperature of 37.4°C, a heart rate of 120 beats/min, a breath count of 35 times/min, and a body weight of 8.75 kg. The child had a round, fleshy face and a loud cry. No jaundice was present in the skin and sclera, and no bleeding or rash was apparent. There was no systemic superficial lymph node enlargement. The bregma was soft and sized 1.0 cm × 1.0 cm. The lip and mouth were not pale and did not exhibit cyanosis, but the throat was congested, and his breath sounds in the lungs were slightly rough with no rattles. The heart rate was regular, and the heart sound was strong with a mild murmur. Significant abdominal distension was observed, and the epigastric veins were obvious. His enlarged liver reached the umbilical level and felt hard, while his enlarged spleen was 3 cm below the costal margin. Limb activity was fair. The simian line was not seen in both hands. The child patient had obvious palmar erythema but no spider angioma. The neurological examination was negative.

Laboratory testing at admission showed low plasma glucose (2.0 mmol/L, reference 3.0–5.9 mmol/L), metabolic acidosis (pH 7.22, PCO_2_ 4.39 KPa, PO_2_ 10.6 KPa, $\mathrm{HC}{O_{3}}^{-}$ 13.0 mmol/L, BE −13.6 mmol/L), elevated lactate (8.7 mmol/L, reference 0.5–1.6 mmol/L), alanine amino transferase (ALT) (161 U/L, reference 10–60 U/L), aspartate amino transaminase (AST) (377 U/L, reference 8–40 U/L), gamma-glutamyl transferase (GGT) (206 U/L, reference 11–50 U/L), serum uric acid (467 µmol/L, reference 208–428 µmol/L), and triglycerides (6.82 mmol/L, reference 0–1.7 mmol/L), and positive results for both blood and urine ketones. Whole blood cell count, plasma total bile acid, total cholesterol, coagulation, urea and creatinine, thyroid function test, creatine kinase (CK)–MB, and blood ammonia were unremarkable. Blood tandem mass spectrometry showed elevation of a variety of acyl carnitines (Table 1), indicating multiple acyl-coenzyme A dehydrogenase deficiency or secondary liver damage. Urine gas chromatography mass spectrometry showed elevated levels of dicarboxylic acids and 3-hydroxyl dicarboxylic acids (Supplementary Table S2). Chest radiography showed that the texture of two lungs was increased, thickened and obscure. The intestines were in the left middle and lower abdomen with a visible twist and grinding-like appearance and tubular inflation. Ultrasonic B mode images showed that the morphologies of the liver and spleen were enlarged, and the liver was 60 mm below the ribs. No intrahepatic bile duct expansion, hepatic venous system, or common bile duct expansion was observed, and the two kidneys were normal in shape and size. The echocardiography results were normal.

**Table 1:** Results of blood tandem mass spectrometry.

Test index	Results (µmol/L)	Prompting	Reference Value (µmol/L)
alanine	344.60	↑	60.00–300.00
glutamic acid	211.64	↑	45.00–200.00
ornithine	10.26	↓	15.00–80.00
acetyl-L-carnitine	72.81	↑	6.00–30.00
3-hydroxy butyryl carnitine	0.77	↑	0.02–0.35
caproyl carnitine	0.33	↑	0.01–0.15
caprylyl carnitine	0.44	↑	0.01–0.30
octene acyl carnitine	0.65	↑	0.03–0.50
suberoyl carnitine	0.06	↑	0.00–0.06
lauroyl carnitine	0.29	↑	0.02–0.20
nutmeg acryloxyethyl carnitine	0.36	↑	0.01–0.30
palmitoyl carnitine	2.33	↑	0.30–2.00
palm enoyl carnitine	0.28	↑	0.02–0.20
18-carbonyl carnitine	1.54	↑	0.20–1.20
18-carbon enoyl carnitine	2.52	↑	0.30–1.80
18-carbon di enoyl carnitine	0.71	↑	0.05–0.60
3-hydroxyl-18-carbonyl carnitine	0.04	↑	0.00–0.03

## Results

### Molecular genetics

**Figure 1:**
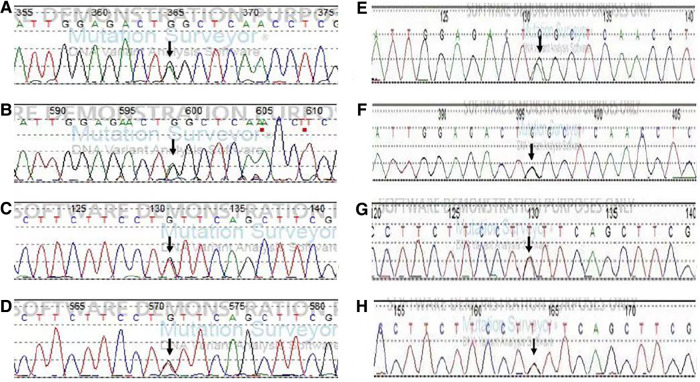
Sequencing results of the G6PC in the patient and his parents. Note: The mutation sites are indicated by arrows. (**A**) c.209G > A in exon1 by forward sequencing in the patient. (**B**) c.209G > A in exon1 by reverse sequencing in the patient. (**C**) c.648G > T in exon 5 by the forward sequencing in the patient. (**D**) c.648G > T in exon 5 by reverse sequencing in the patient. (**E,F**) c.209G > A in exon 1 from his father (by forward and reverse sequencing). (**G,H**) c.648G > /T in exon 5 from his mother (by forward and reverse sequencing).

Because the changes in blood carnitine and urinary organic acid spectra suggested MADD, whereas the clinical and routine biochemical changes indicated GSD Ia, Sanger sequencing (primers showed in Supplementary Table S1) on coding and flanking sequences of G6Pase and ETFDH from periphery blood sample was ordered for the patient and his parents. The variants nomenclature follows the Human Genome Variation Society guidelines (www.hgvs.org/mutnomen) and refers to the G6Pase gene coding sequence (transcript: NM_000151.4). Coding and flanking sequences of the G6Pase gene detected two variants: one nonsense variant c.209G > A (p.Trp70Ter) was paternally inherited and another synonymous variant c.648G > T (p.Leu216Leu) was maternally inherited (Figure 1). c.209G > A was classified as pathogenic variant according to ACMG guideline for a very low frequency in gnomAD (1/140,200, PM2_ Supporting), predicting to form a stop code that causes premature termination of the transcript (PVS1), and segregated with a phenotype or disease in multiple affected family members and multiple families (PP1_ Strong) (2–4). c.648G > T was classified as pathogenic variant according to ACMG guideline for a very low frequency in gnomAD (11/121,406, PM2_ Supporting), a pathogenic variant detected in trans (PM3), segregated with a phenotype or disease in multiple affected family members and multiple families (PP1_ Strong) (2, 5) and altered splicing by producing an aberrant transcript that eliminated 91 nucleotides deletion in exon 5 resulting in a premature termination (PS3) (5). Sequencing all exon and flanking sequences of the ETFDH gene revealed no variants in the proband.

### Management

Based on the clinical manifestations, biochemical changes and blood and urine mass spectrometry results, we suspected that the child had GSD Ia or MADD; therefore, oral uncooked cornstarch and high-dose vitamin B2 were administered with other symptomatic and/or supporting management. During hospitalization, the child experienced secondary rotavirus enteritis and acute otitis media with episode hypoglycemia and transaminase fluctuations. After 20 days, the child was discharged with a stable body temperature, no cough, stable blood glucose levels, corrected metabolic acidosis, negative blood and urine ketones and normal blood lactate and uric acid levels. A number of biochemical parameters were significantly improved (ALT 87 U/L, AST 155 U/L, GGT 178 U/L, triglycerides 3.14 mmol/L).

After discharge, the patient continued to receive oral uncooked cornstarch while gradually stopping vitamin B2 supplementation. The reexamination results of blood tandem mass spectra and urine gas mass spectra (Supplementary Table S2) were normal. At the age of 12 months, the patient's weight was 11 kg, and he was in good general condition without growth retardation. The blood biochemical indices were as follows: blood glucose 5.7 mmol/L, ALT 84 U/L, AST 82 U/L, GGT 66 U/L; serum uric acid 243 µmol/L, triglycerides 5.0 mmol/L, total cholesterol 3.1 mmol/L. Ultrasonic B mode images showed an enlarged liver morphology, regular capsule, fine and enhanced liver echo with nonuniform distribution, the liver 34 mm below the costal margin, and a slightly large spleen. When the child was 29 months old, his weight was 15.3 kg, and his body length was 92.6 cm. The child was followed up to 33 months of age, and the blood biochemical indices returned to normal as follows: blood glucose 3.79 mmol/L, ALT 22 U/L, AST 27 U/L, GGT 22 U/L; serum uric acid 370 µmol/L, triglycerides 2.42 mmol/L, total cholesterol 4.57 mmol/L. Ultrasonic B mode images showed an enlarged liver morphology, smooth surface, uniform echo, clear blood vessels, and normal sized gallbladder, pancreas, spleen and kidney.

## Discussion

 GSD Ia shares some clinical manifestations with MADD such as liver enlargement, hypoglycemia and acidosis. But these two diseases should be managed differently and carries a different prognosis. The treatment of GSD Ia mainly consists of oral uncooked cornstarch to prevent the occurrence of hypoglycemia (6). The main treatment for MADD consists of a low fat, low protein diet with supplementation of riboflavin and carnitine, which can significantly improve the symptoms and biochemical indexes, and the prognosis is good if the diagnosis is timely and early preventive treatment is given (7–10). Therefore, the differential diagnosis of GSD Ia from MADD is very important.

The child patient we reported was suspected to have MADD due to changes in mass spectra; however, he was eventually genetically diagnosed as having GSD Ia. MADD is a relatively common congenital metabolic disorder of fatty acid oxidative metabolism. It has been reported that approximately 62% of patients with fatty acid oxidation metabolism disorders in China suffered from MADD (11), and ETFDH defects are the most common form of this disease (12, 13). According to the clinical manifestation, MADD can be divided into three types, as follows: type I: newborn with congenital anomalies (such as polycystic kidney, cardiomyopathy, and facial deformity); type II: newborn without congenital anomalies; and type III: late-onset type or light type.

The clinical manifestations of MADD in children include myasthenia, enlarged liver, paroxysmal hypoglycemia, hypertrophic cardiomyopathy and rash during onset of the disease, while laboratory biochemical tests may show increased AST, ALT, CK, CK-MB and lactate dehydrogenase (LDH) levels and metabolic acidosis. Increased CK and CK-MB can be seen in MADD when cardiac muscle and skeletal muscle are involved. Mass spectrometry was characteristic changes in MADD. Increased levels of short-chain, middle-chain and long-chain acyl carnitines by blood tandem mass spectrometry and possible dicarboxylic aciduria of ethyl malonic acid, glutaric acid, adipic acid and pimelic acid by urine gas chromatography mass spectrometry may be observed (14).

In recent years, with the extensive development of mass spectrometry, blood tandem mass spectrometry and urine gas chromatography mass spectrometry play an important role in the screening and diagnosis of inherited metabolic diseases (15). However, different genetic metabolic diseases may share similar metabolite fluctuations, and the diseases may be misdiagnosed if mass spectrometry is only relied on for diagnosis. Therefore, the confirmation of a MADD diagnosis requires further genetic evidence. Riboflavin-reactive MADD can typically be cured, especially for patients with the ETFDH variant, thus highlighting the important role of genetic testing in these patients (16).

Based on the combined results of clinical manifestations, blood tandem mass spectrometry and urine gas chromatography mass spectrometry of the patient, MADD was a diagnosis that needed more investigation. So we treated the patient with oral vitamin B2 at the beginning. However, the normal serum CK level and no pathogenic/likely pathogenic variants in exons of ETFDH in this patient did not support the diagnosis of MADD. More importantly, the abnormal results of the blood tandem mass spectrometry and urine gas chromatography mass spectrometry normalized quickly after the hypoglycemia was corrected in the child, even after the discontinuation of vitamin B2 administration, which make the diagnosis of MADD very unlikely. Up to now, no signs of MADD developed.

The main clinical manifestations of GSD Ia are liver enlargement, fasting hypoglycemia, short stature, obesity, etc. Blood biochemical examination showed elevated blood lipids, metabolic acidosis, hyperlactatic acidemia, elevated uric acid. GSD Ia does not involve the heart and muscle, so CK and CK-MB are mostly normal. In reviewing the history of the child patient who showed a significant enlarged liver, elevated transaminases, hypoglycemia, lactic acidosis, hyperlipidemia, and hyperuricemia, it was found that these symptoms were all typical clinical manifestations of GSD. However, because the mass spectra of the child showed increased blood acyl carnitines and dicarboxylic aciduria, which are not reported in GSD cases, make the diagnosis process more complicated. We tested the G6Pase gene of the patient and his parents and found compound heterozygous single-base variants in the G6Pase gene in the patient. c.209G > A was derived from the father, leading to amino acid 70 being changed from tryptophan to the termination codon (p.Trp70Ter). Furthermore, c.648G > T (p.Leu216Leu) from the mother, was reported as the most common pathogenic variant in Chinese GSD Ia patients (17). The child was finally diagnosed with GSD Ia. Thus, for patients with increased blood acylcarnitines and dicarboxylic aciduria as evidenced by mass spectrometry, the possibility of GSD should also be considered during differential diagnosis.

The abnormal mass spectrum change in this child was the main reason for the misdiagnosis. No similar report has been published to our best knowledge. Why did the results of mass spectrometry in this patient show similar changes to MADD? The exact mechanism is unknown. However, the following speculations can be made. G6Pase is a key enzyme for glycogenolysis and glucose production, which plays an important role in maintaining normal blood glucose levels. When there is a congenital defect in G6Pase, glycogen cannot break down into glucose, leading to hypoglycemia. Hypoglycemia causes fat mobilization, which produces large amounts of fatty acids, Oxidative metabolism of fatty acids requires multiple acyl-coenzyme A dehydrogenase. When fatty acids are produced too much, these enzymes may be relatively inadequate. In addition, stress may affect the activity of these enzymes, such as fever, fatigue, hunger, etc.. In this case, the child patient was born with hypoglycemia, indicating that he was in serious condition. After fat mobilization, a large amount of fatty acids were produced. When levels are relatively insufficient and intermediate metabolites are increased, increased blood carnitine levels and dicarboxylic aciduria result, with even more energy needed during infection. Because mass spectrometry is not routinely ordered in patients with suspected GSD Ia, we don't know whether these metabolic changes occur in other GSD Ia patients with severe hypoglycemia.

In summary, GSD Ia can mimic the abnormal mass spectrum results as well as the typical clinical manifestations of MADD. Blood tandem mass spectrometry and urine gas chromatography mass spectrometry play an important role in the diagnosis of inherited metabolic diseases; however, these diseases may be misdiagnosed if simply relying on mass spectrometry. Therefore, genetic testing is currently very important for confirming the diagnosis of inherited metabolic diseases. One limitation of this paper is that the G6Pase genes and ETFDH genes were not completely sequenced. One reason is that the c.209G > A or c.648G > T variant that cause MADD are very rare and another is that the rapid and persistent normalization of blood acyl carnitine and urinal organic acid profiles without specific administration is very unlikely.

## Data Availability

The datasets presented in this study can be found in online repositories. The names of the repository/repositories and accession number(s) can be found in the article/**Supplementary Material**.
